# Epidemiology of dementia in Central Africa (EPIDEMCA): protocol for a multicentre population-based study in rural and urban areas of the Central African Republic and the Republic of Congo

**DOI:** 10.1186/2193-1801-3-338

**Published:** 2014-07-03

**Authors:** Maëlenn Guerchet, Pascal Mbelesso, Bébène Ndamba-Bandzouzi, Sophie Pilleron, Ileana Desormais, Philippe Lacroix, Victor Aboyans, Pierre Jésus, Jean-Claude Desport, Achille E Tchalla, Benoît Marin, Jean-Charles Lambert, Jean-Pierre Clément, Jean-François Dartigues, Pierre-Marie Preux, the EPIDEMCA group

**Affiliations:** Tropical Neuroepidemiology, Faculty of Medicine, INSERM UMR 1094, 2 rue du Docteur Marcland, 87025 Limoges, France; Institute of Neuroepidemiology and Tropical Neurology, University Limoges, School of Medicine, CNRS FR 3503 GEIST, Limoges, France; King’s College London, Centre for Global Mental Health, Institute of Psychiatry, Health Service and Population Research Department, London, UK; Department of Neurology, Brazzaville University Hospital, Brazzaville, Republic of Congo; Department of Neurology, Amitié Hospital, Bangui, Central African Republic; Department of CardioVascular Surgery, CHU, Limoges, France; Department of Cardiology, Dupuytren University Hospital, Limoges, France; Department of Nutrition, CHU, Limoges, France; EA 6310, Disability, Activity, Aging, Autonomy and the Environment (HAVAE), Limoges, France; CHU, Department of Medical Information & Evaluation, Clinical Research and Biostatistic Unit, Limoges, France; INSERM U744, Institut Pasteur de Lille, Lille, France; Hospital and University Federation of Adult and Geriatric Psychiatry, Limoges, France; INSERM U897, Victor Segalen Bordeaux II University, Bordeaux, France

**Keywords:** Ageing, Dementia, Alzheimer’s Disease, Prevalence, Risk Factors, sub-Saharan Africa

## Abstract

**Background:**

The worldwide population is ageing and the proportion of elderly aged 60 and over is expected to dramatically rise in Low and Middle Income Countries (LMIC). The epidemic of dementia will not spare those countries, where the largest increases in numbers of people affected are estimated. Besides, dementia is still understudied in sub-Saharan Africa (SSA) compared to other regions. This paper describes the protocol for the ‘Epidemiology of Dementia in Central Africa’ population-based study, which aims at estimating the prevalence of dementia in two countries of Central Africa and investigating possible risk factors.

**Methods/Design:**

A multicenter population-based study was carried out in Central African Republic and Republic of Congo between 2011 and 2012 including both urban and rural sites in each country. Around 2000 participants aged ≥65 years old were interviewed in total using the Community Screening Interview for Dementia (CSI-D), the GMS-AGECAT and the CERAD’s 10-word list. Elderly with low performance to the cognitive part of the CSI-D (COGSCORE ≤ 24.5) were then clinically assessed by neurologists and underwent further psychometrical tests. DSM-IV and NINCDS-ADRDA criteria were required for dementia and Alzheimer’s disease (AD) diagnoses respectively. The algorithmic 10/66 dementia diagnosis was also determined. Petersen’s criteria were required for the diagnosis of Mild Cognitive Impairment. Sociodemographic, and environmental factors including vascular, nutritional, biological, psychosocial and lifestyle factors were collected in each setting in order to investigate factors associated with dementia. Blood sampling was realized to investigate genetic variations that could modify the risk of dementia.

**Discussion:**

For now, no large epidemiological study has been undertaken to compare the prevalence of dementia in both rural and urban areas within SSA countries. This programme will provide further evidence regarding the prevalence of dementia in SSA, and also the possible rural/urban disparities existing with associated factors. Furthermore, the genetics of AD in those populations will be addressed.

## Background

Due to an increase of life expectancy, the worldwide population is ageing. The number of people and relative proportion aged 60 years and over is increasing. However, this demographic transition is faster in low and middle-income countries (LMIC) than it was during the last century in high-income countries (HIC), and the proportion of elderly aged 60 and over is expected to rise from 8% in 2000 to 20% in 2050 in less developed regions (World Population Prospects 2003). The burden of non-communicable diseases, including age-related diseases, is increasing and will add to the existing burden of infectious diseases. The epidemic of dementia is going to affect considerably those LMIC, where 57% of all people with dementia lived in 2010, rising to 71% by 2050 (Prince et al. 2013a). The largest increases in projected numbers of people with dementia are those estimated for Eastern Asia and sub-Saharan Africa (SSA) (Prince et al. 2013b).

Despite an increase in the number of studies on dementia and Alzheimer’s Disease (AD) carried out in LMIC during the last years, there is still a particular dearth in epidemiological studies for some regions of the world, especially Russia, Indonesia, the Middle-East and Africa (Prince & Jackson 2009). While the 10/66 Dementia Research Group estimated dementia prevalence and incidence, studied risk factors and predictors of dementia, disability, dependence and mortality across several LMIC of Latin America (Cuba, Dominican Republic, Puerto-Rico, Peru, Mexico, Venezuela), India and China (Llibre Rodriguez et al. 2008; Jotheeswaran et al. 2010; Sousa et al. 2009; Sousa et al. 2010; Ferri et al. 2011; Albanese et al. 2013; Prince et al. 2012), dementia is still understudied in sub-Saharan Africa. In the 90s in Nigeria, the Indianapolis-Ibadan Dementia Project estimated the age-adjusted prevalence of dementia (according to DSM-IV/ICD-10 criteria) at 2.3% in the Yorubas aged 65 and over living in Ibadan, and the prevalence of AD at 1.4% (Hendrie et al. 1995). These low prevalences of dementia were first confirmed by two population-based studies in Benin, where the age-standardized prevalences of dementia were estimated at 3.3% in a rural area (Guerchet et al. 2009) and 2.9% in Cotonou, the economic capital (Paraïso et al. 2011). Nevertheless, other recent studies from Central and East Africa showed a different picture, reporting age-standardized prevalences of 7.6% and 5.4% respectively in capitals of Central African Republic and Republic of Congo (Guerchet et al. 2010) while the age-standardized prevalence in rural Tanzania was 6.4% (Longdon et al. 2013). In contrast with Latin American countries, it is less clear whether urban living is associated with dementia in other regions of the world (Kalaria et al. 2008). For now, no multicentre study using the same protocol has been undertaken to compare the prevalence of dementia in both rural and urban areas within SSA countries.

Determinants of dementia risk, modifiable or not, are suggested to impact across the life course similar to other chronic conditions of late life (Prince et al. 2014). Ageing is the main risk factor for dementia, with a prevalence doubling with every 5.5 to 6.7 years increment in age over the age of 60 (Prince et al. 2013a). Vascular factors are also important contributors to dementia risk (Barnes & Yaffe 2011), with evidence showing associations between dementia and midlife hypertension (Qiu et al. 2005; Kennelly et al. 2009), diabetes (Biessels et al. 2006; Profenno et al. 2010), midlife obesity (Barnes & Yaffe 2011), elevated midlife cholesterol (Solomon et al. 2009), physical inactivity (Hamer & Chida 2009) and smoking (Anstey et al. 2007; Peters et al. 2008). Among all psychosocial factors, history of depression (Jorm 2001; Ownby et al. 2006) is a robust possible risk factor. Many other aspects are also investigated (diet, female reproductive health, environmental factors, cognitive activity or traumatic brain injury) often showing conflicting results.

However, once again, the evidence is mostly issued from studies carried out in HIC or any other continent than SSA, and data for this region are scarce. For now, common factors like age or female sex have been associated with cognitive impairment or dementia in Nigeria, Benin and Central Africa (Guerchet et al. 2010; Ochayi & Thacher 2006; Hall et al. 1998; Ogunniyi et al. 2000; Ogunniyi et al. 2006; Gureje et al. 2006), mainly in cross-sectional studies. A low Body-Mass Index (<18.5 kg/m^2^), indicating undernutrition, was also retrieved in both Nigeria and Central Africa (Ochayi & Thacher 2006; Guerchet et al. 2012), while other associations were reported in Nigeria only (lifetime history of alcohol use, living with others) (Ogunniyi et al. 2000; Gureje et al. 2006) or Central Africa only (hypertension, depressive symptoms, death of a parent before age of 16, recent moving) (Guerchet et al. 2012). In the same populations, education was not significantly associated with dementia in multivariate analysis (Ochayi & Thacher 2006; Hall et al. 1998; Ogunniyi et al. 2000; Ogunniyi et al. 2006; Gureje et al. 2006). However, a small proportion of the participants had formal education beyond primary school in these studies.

The attributable genetic risk in common forms of dementia and particularly AD, is exceptionally high (~80%) amongst common multifactorial ageing diseases, e.g. well above those reported for type II diabetes (~40%) or Parkinson's disease (~30%) (Gatz et al. 2006). Even if this evaluation has been done in populations from Caucasian origins, it is reasonable to assume that it is the same for populations from other origins such as subjects of African ancestry. However, such a postulate raises the following question: are the recognized genetic risk factors in Caucasians (e.g, monogenic mutations, APOE and GWAS defined-genes) similar of the ones involved in Africans (European Alzheimer's Disease Initiative (EADI) et al. 2013; Cruchaga et al. 2014; Guerreiro et al. 2013)?

Most of the information available are indirect and concern subjects of African-American ancestry for whom APOE and several GWAS-defined genes have been associated with AD risk (Reitz et al. 2013; Logue et al. 2011). Very few studies were able to address AD genetics in Africans. To date, a PS1 mutation has been reported in a large family of indigenous Southern Africans (Heckmann et al. 2004) and the ϵ4/ϵ4 genotype has been associated with an increase in AD risk in the Nigerian Yoruba population (Hendrie et al. 2014).

### Aims of the EPIDEMCA programme

1. To estimate and compare the prevalence of dementia and related syndromes in urban and rural areas in Central Africa (Central African Republic and Republic of Congo), and to study the neuropsychological profiles of demented and non-demented subjects,
2. To investigate factors associated with dementia and cognitive disorders: sociodemographic, environmental factors including vascular, nutritional, lifestyle and biological factors,
3. To determine if genetic variations can modify the risk of dementia in African populations,
4. To create a biobank from native elderly Africans in order to carry out further biological, genotypic and phenotypic researches in the future.

## Methods/Design

### Study design

Cross-sectional two-phase surveys were conducted between November 2011 and December 2012 in a sample of 2002 people aged 65 years old and over; with a sample size around 500 participants in each of the 4 settings (Table 1), with an identical data collection protocol in the 4 settings for the sake of comparability (dementia screening and diagnosis, demographics, physical assessment, and risk factor questionnaire). Frozen blood plasma and DNA will be available from each setting and will be saved for further biochemical and genetic analyses.

**Table 1:** **EPIDEMCA population-based survey, Central Africa, 2011-2012**

Country	Study area	Sample	Interviews completed	Response rate%	Blood sample	Lost-of follow-up	Start/End
Central African Rep.	Nola (rural)	501	473	94.41	393	66	Nov. 2011/Feb. 2012
	Bangui (urban)	514	500	97.28	441	48	Jan. 2012/March 2012
Rep. of Congo	Gamboma (rural)	561	529	94.30	836	93	Aug. 2012/Dec. 2012
	Brazzaville (urban)	537	500	93.11	409	17	Sept. 2012/Nov. 2012

### Settings

**Figure 1:**
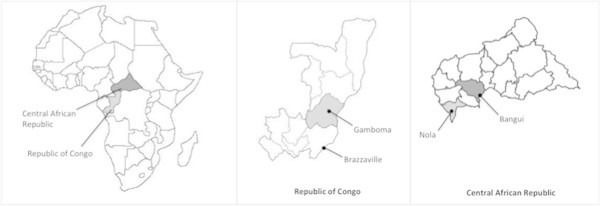
**Localisation of the Republic of Central Africa and Republic of Congo, and rural and urban settings within each country.**

Four settings were investigated in two LMIC: the Central African Republic (CAR) and the Republic of Congo (ROC) (Figure 1). In each country, one urban area (respectively Bangui and Brazzaville) and one rural area (Nola and Gamboma) were included. Urban areas were both capitals, and the biggest cities, of those countries. Rural areas were defined by a lower population density and traditional agrarian lifestyle.

The Central African Republic is a landlocked low-income country (The World Bank, Data, Central African Republic). According to the last national census (Recensement Général de la Population et de l’Habitat 2003), its population includes 2.6% aged 65 years and over, and the life expectancy at birth is 48.3 years (The World Bank, Data, Central African Republic). Bangui is located on the side of Oubangui river, in the South of the country, and its population is estimated at 622 771 inhabitants. The city is divided in 8 main administrative subdivisions over 67.0 square-kilometres. Nola, the rural site located in the Sangha-Mbaéré prefecture, is situated 422 kilometers south-west from Bangui, with a total population of 7419 inhabitants. Smaller villages within the 10 kilometres radius from Nola were also included into the rural CAR setting.

The Republic of Congo is a lower-middle income country (The World Bank, Data, Republic of Congo). Its population aged 65 years old was estimated at 3.2% of the total population in the last national census (2007) (Recensement Général de la Population et de l’Habitat 2007 2010). Life expectancy at birth is 51.1 years (The World Bank, Data, Republic of Congo). Brazzaville is located on the side of the Congo River, in the south of the country, and its population is estimated at 1 373 382 inhabitants. The city is divided in 9 main administrative subdivisions, over 263.9 square-kilometers. Gamboma, a rural setting in the Plateau Region, is situated 314 kilometres north from Brazzaville, with a total population of 18 514 inhabitants.

Albeit neighboring countries, CAR and ROC show some disparities (Table 2) (United Nations Development Program 2013). The main industries of ROC are linked to oil and wood; other industries are poorly developed and of only local importance. Forestry and mining are the only developed industries in CAR; there are essentially no others. Brazzaville is overall more urban, with increased development of a Western way of life and higher socio-economic conditions as compared to Bangui.

**Table 2:** **Characteristics of the Central African Republic and the Republic of Congo**

	RCA	ROC
Surface (km^2^)	623 000	342 000
Number of inhabitants	3 895 139	3 697 490
Capital	Bangui	Brazzaville
Density population (inhab/km^2^)	7.2	12.4
Rural population	62%	35%
Human development indice	0.352	0.534
Human development indice rank 2013 (/186)	180	142
Gross domestic product per capita (PPP US$)	722	2 934
Life expectancy at birth (years)	49.1	57.8
Poverty headcount at national poverty line	62%	47%

*GDP: Gross domestic product*.

http://www.who.int/countries/en/ *and* http://hdr.undp.org/en/statistics/ *.*

### Sampling and sample size

Sampling methods differed between urban and rural areas. To be representative of Bangui and Brazzaville inhabitants, a random sampling proportional to the size of each main subdivision of the city was realized in urban sites. For each main subdivision, a district has been randomly chosen. Then, the procedure was the same in each district: door-to-door survey was conducted in a random direction, starting from the district chief’s house, until the number of participants allocated to each subdivision was reached. If the number of participants was not reached in the first district, the procedure was applied in another random district belonging to the same subdivision.

In rural areas, a door-to-door approach was preferred due to logistical and financial constraints. Each district constituting the area was investigated. In case of absence of inhabitants during the day of screening, houses were revisited at least twice to check the presence of old people.

For both types of settings, every house visited was marked by the investigators (name of the study, number of the house, participant and investigator) with chalk, just above the door, ensuring exhaustive coverage, even in the absence of addresses.

The only exclusion criteria were refusal or the presence of severe comorbidities precluding the interview and/or cognitive testing in absence of an informant.

Precision calculations indicated that a sample of 456 people in each site would allow estimation of an expected dementia prevalence of 5% with a precision of +/- 2% (EpiInfo 6.04, Epiconcept). We then aimed at including about 500 elderly in each setting.

The sample of elderly included in this study was aged between 65 and 99 years (maximum 93 in Nola and Bangui, 99 in Gamboma, and 95 in Brazzaville), with a mean age of 73.3 ± 6.7 years (respectively 72.8 ± 6.5, 72.6 ± 6.4, 74.0 ± 6.9 and 73.6 ± 6.7 years in each setting). Women represented the highest proportion of our population, with a global sex-ratio (M/F) of 0.63, ranging from 0.54 to 0.69 in CAR and of 0.64 for both areas in ROC. A small proportion (5.4%) of the participants reported to live alone (from 1.6% in Bangui to 6.8% in Nola), and more than two-third (68.8%) did not receive any formal education (75.4% in Nola and 63.3% in Bangui, 82.0% in Gamboma, and 54.1% in Brazzaville).

### Preparation

Each coordinator collaborated on neuroepidemiological studies for several years and has already been involved in previous studies on dementia (Guerchet et al. 2010; Mbelesso et al. 2012). They participated to design the study protocol before applications for funding. Conference calls and live meetings were held regularly during the year prior to the study.

In each country, Universities and Ministries of Health, Research and Public Health were informed and supported the study. Prefectures, town halls and sanitary districts received information about the aims and schedule of the study. District chiefs were visited prior to start the screening in order to increase awareness and population acceptance. Messages were broadcasted on a local radio in Nola (CAR) and disseminated by town criers in Gamboma (ROC) and Bangui (CAR).

### Interviews and measures

Assessments were translated into the relevant local languages: Sango in CAR, Lingala, Kituba and Lari in ROC. These translations were done from French to local languages, then from local languages to French in order to observe translation consistency.

The first phase was conducted in the participant or their relative’s homes, including the following assessment: cognitive and physical assessment, demographics and risk factor questionnaire. After the identification of participants at risk of dementia through the questionnaires, the second phase was conducted few weeks later (between 3 and 14), at the closest hospital or community health centre, by neurologists in presence of selected participants and an informant, most of the time one of their relatives.

1. ***Epidemiological assessements***
  The participants, or informant in case of communication difficulties due to dementia, poor physical condition, extreme fatigue, hearing or oral problems, have provided this information.

#### Sociodemographic status

Age was ascertained by official documents (national identity cards, passports, birth certificates). In case of absence of these documents or discrepancy between documents and participants reports, age was estimated using two historical landmarks in each country or through a local event calendar, using a validated method (Ogunniyi & Osuntokun 1993; Paraïso et al. 2010). Age was ascertained from an informant if none of those methods was successful.

Marital status, living circumstances, education (no formal education, primary, secondary or higher education, certificate of primary school validated or not), literacy, and ethnic group were collected. Further information about rural or urban residence across life course, current occupation, and past occupation was sought.

Participation to religious ceremonies or other social activities (playing games and meeting with friends) was recorded, as well as their participation to children’s education. Indications on happiness of each participant were collected using one section of the Center for Epidemiologic Studies Depression Scale (Radloff 1977).

#### Lifestyle and cardiovascular risk factors

Tobacco and alcohol use were investigated through self-report. Mode of consumption (smoked, quid, snuff tobacco), volume and frequency, and lifetime smoking (never, ever or current smokers) were recorded. Alcohol consumption was described (alcohol type, frequency and units of alcohol per week).

Diet was investigated. A food frequency questionnaire asked if people ate their fill or not, whether the food was all consumed, and if there were any dietary restrictions. The number of meals per day was recorded, as were the reasons for people eating fewer than three. The frequency of consumption over the past 3 days was recorded for dairy products, fruits, vegetables, starches, legumes, oil seeds, meats or fishes, eggs, and sweet foods.

Current physical activity was estimated using a threshold of at least 150 minutes of walking or cycling in the past week (World Health Organization 1984a).

2. ***Clinical assessments***

#### Health status

Self-reported comorbidities were investigated through the Charlson’s weighted comorbidity index (Charlson et al. 1987). Self-reported diagnosis concerned stroke, diabetes, hypertension, and treatments for these conditions, using the following questions: ‘Have you ever been told that you had…? Were you started on treatment? Are you still on treatment?’. Familial history of dementia and previous head injury were also investigated.

A full direct physical assessment was realized including the following measures.

- Height (expressed in centimetres, measured by a carpenter meter along a flat surface), weight (expressed in kilograms measured on SECA® mechanical scales). Height was estimated using the Chumlea formula based on the heel-knee distance if the height was impossible to measure (participant unable to stand upright) (Chumlea et al. 1998). Body Mass Index (BMI) was calculated by dividing weight in kilograms by height in metres squared (kg/m^2^) and categorized according to the WHO recommendations: BMI <18.50 is considered underweight, BMI between 18.50 and 24.99 is considered normal range, a BMI between 25.00 and 29.99 overweight, and a BMI ≥ 30.0 defines obesity (World Health Organization 1984b).
- Waist circumference (measured in cm, midway between the lower rib margin and iliac crest) and hip circumference (measured in cm, at level of the highest circumference by including buttock). The waist-to-hip ratio (WHR) was then calculated by dividing waist circumference by hip circumference. The presence of abdominal obesity was determined following the recommended waist circumference thresholds for abdominal obesity in sub-Saharan Africans (Alberti et al. 2009).
- Mid-upper arm circumference (MUAC, measured at the nearest mm, on the right arm, midway between acromion and olecranon), triceps skinfold thickness (TST, measured on right arm three times to the nearest 0.2 mm according to Lohman standard procedures (Lohman et al. 1988) with an Harpenden calliper). Arm muscle circumference (AMC, cm), representing muscle mass, will be calculated using the following formula: AMC = MUAC (cm) - (0.314 *TST (mm)) (Gurney & Jelliffe 1973).
- Leg length (measured from the iliac crest down to the lateral malleolus on the right leg, in centimetres) and skull circumference (measured just above the participant’s eyebrows and round to the occipital pole at the back of the skull, in centimetres).
- Pulse rate, systolic and diastolic resting blood pressure (two measures for each arm) were recorded. Hypertension was defined in case of self-reported ongoing treatment and/or when systolic blood pressure (SBP) at rest was ≥140 mmHg and/or diastolic blood pressure (DBP) was ≥90 mmHg (World Health Organisation 2013).
- Ankle-Brachial Index (ABI): Doppler ultrasound (Super Dopplex II, Huntleigh Technology PLC, Luton, UK) was used to measure SBP in the posterior tibial and dorsal pedis arteries bilaterally. The measurement protocol and ABI calculation were performed according to the American Heart Association guidelines (Aboyans et al. 2012). Peripheral artery disease will be defined as an ABI ≤0.90.
- Capillary blood glucose level (Accu-Chek® Performa, Roche). Diabetes was defined according to self-reported diabetes or in case of elevated blood glucose level, above 126 mg/dL if the fasting period > 2 hours or above 200 mg/dL in non-fasting participants (World Health Organization 2006).

#### Frailty

The SOF index (Study of Osteoporotic Fracture) was calculated based upon three components: self-reported unintentional weight loss, inability to rise from a chair five times without the use of arms, and self-reported low energy level. Frailty status was defined as robust (0 components), prefrail (previously referred to as “intermediate”, 1 component), and frail (≥2 components) (Kiely et al. 2009). The walking speed (over a 4.5 metre distance) was also recorded.

3. ***Psychological assessments***
  The Community Screening Interview for Dementia (CSI-D) was performed with each participant (Hall et al. 1993), allowing the calculation of the COGSCORE (incorporating the CERAD animal naming verbal fluency) and the RELSCORE (providing evidence of cognitive and functional decline reported by an informant). As previous studies (Guerchet et al. 2009; Paraïso et al. 2011; Guerchet et al. 2010) highlighted the difficulty to perform CSI-D praxies items for subjects who have no formal education, the last items (requiring writing skills) were replaced by two-items from the Stick Design Test (Baiyewu et al. 2005).
  A structured clinical mental state interview, the Geriatric Mental State version B3 (GMS-AGECAT) (Copeland et al. 1986) identifying depression, anxiety and psychosis, and the modified CERAD 10 word list learning task with delayed recall (Ganguli et al. 1996) were added to this cognitive battery.

#### Diagnoses of MCI, Dementia, Alzheimer’s disease

**Figure 2:**
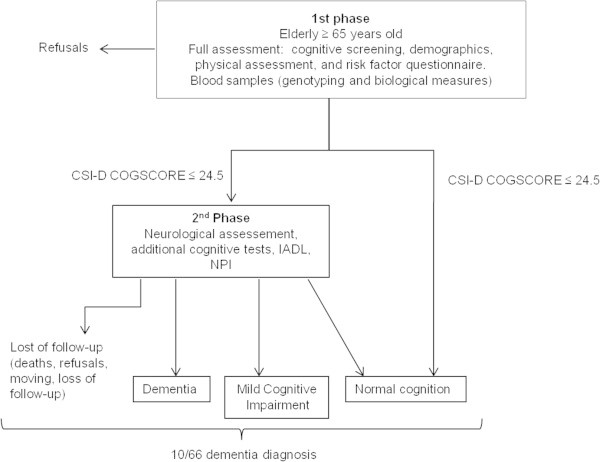
**Flow chart of the EPIDEMCA study, Central Africa, 2011-2012.**

All subjects who obtained a poor performance to the CSI-D (COGSCORE ≤ 24.5, sensitivity = 93% and specificity = 82% in the EDAC study, personal data) were convened to a further clinical assessment with a neurologist (second phase) (see Figure 2). Neurological assessments were completed by additional cognitive tests: the Free and Cued Selective Reminding Test (Grober et al. 1988) (oral version with image), Zazzo’s cancellation task (Zazzo 1974) and Isaac’s Set Test of verbal fluency (Isaacs & Kennie 1973). The questionnaire was administered during a face-to-face interview of the subject and of an informant.

Careful description of daily living activities particularly leisure, other social activities, Instrumental Activities of Daily Living with the same level of precision as the Lawton scale (Lawton & Brody 1969) but adapted to the African context, occupational activities, social habits, and orientation skills were investigated in order to evaluate the dependence.

Diagnosis of dementia was made according to the DSM-IV criteria (American Psychiatric Association 2003) and according to the clinical criteria proposed by the NINCDS-ADRDA (National Institute of Neurological and Communicative Disorders and Stroke and the Alzheimer’s Disease and Related Disorders Association) for AD (McKhann et al. 1984). Diagnosis for other types of dementia (especially vascular dementia) was based on history of stroke and other clinical arguments. Petersen’s criteria were used for Mild Cognitive Impairment (Petersen 2004). Experienced neurologists reviewed all medical records and performances to tests, and consensus on the diagnosis was obtained. Subjective memory complaints were recorded with the ‘Questionnaire de Plainte Cognitive’ (Thomas Antérion et al. 2003) and the severity of dementia evaluated by the Clinical Dementia Rating Scale (Morris 1993).

Behavioural and Psychological Symptoms of Dementia were investigated through the NeuroPsychiatric Inventory (NPI) (Cummings et al. 1994).

The 10/66 dementia diagnosis was determined later, using the 10/66 validated dementia computerized algorithms based upon the CSI-D, the CERAD 10 word list learning and animal naming tests, and the Geriatric Mental State B3 (Prince et al. 2008).

#### Stressful life events

Stressful psychosocial factors were investigated for three different life periods as already described by Persson & Skookg (Persson & Skoog 1996): before 16, between 16 and 64, and after 65 years old. Eighteen life events were looked for: 5 before the age of 16 (death of a parent, divorce of parents, growing up with one parent, different guardians, extreme poverty), 5 between 16 and 64 years old (death of a spouse, death of a child, serious illness in a child, shift or piece work and arduous manual work), and 8 from 65 years of age (death of spouse, physical illness in spouse, mental illness in spouse, death of a child, serious illness in a child, death of siblings or friends, change of residence and financial status deterioration).

#### Dependent personality disorders

The presence of dependent personality disorders (DPD, DSM-IV Axis II) was screened through the dependent personality domain of the Personality Diagnostic Questionnaire –4 plus (PDQ4+) (Hyler 1994). Dependent personality disorders were present if at least five items out of 8 were responded positively.

4. ***Biological assessments***

#### Blood sample protocol

Blood samples were sought from all participants. After the interview and the physical assessment, dedicated nurses performed a phlebotomy for consenting participants. Blood was drawn on two 10 ml polypropylene EDTA tubes for DNA extraction and biological analyses. Samples were registered in a database, centrifuged and aliquoted within few hours (to avoid degradation due to high temperatures), and then transferred to a laboratory to be frozen at -20°C or -80°C. Plasma and buffy-coat aliquots were stored at the Pasteur Institute of Bangui and the National Laboratory of Public Health in Brazzaville before dry-ice shipping to the University of Limoges, where the biobank is located.

#### Biological assessments

Total cholesterol and C - reactive protein (CRP) dosages were performed on plasma aliquots in the Biochemistry department of the Limoges University Hospital using standard procedures (on Cobas automaton). Hypercholesterolemia was then defined when above 5.3 mmol/L and a high CRP level, an inflammatory marker, was considered when above 5 mg/L.

#### Genotyping

DNA was extracted in the Inserm U744 unit (Pasteur Institute, Lille, France) using standard procedures. Association of APOE and GWAS-defined genes with AD risk will be assessed in order to evaluate whether genetic risk factors in Caucasian are also involve in individuals of African ancestry. We acknowledge that the statistical power of our study might be restricted according to the magnitude and level of association of most of these genes with AD risk in Caucasian. However a recent work indicated that variants in ABCA7 might have a stronger impact in African-Caucasians than the one previously observed in Caucasians. Genetic analyses in our samples might thus allow to point out genes with particularly high impact in Africans and thus suggest specific pathophysiological pathways to be targeted for drug designs in these populations.

### Resources/training/quality control

Every country had a local project coordinator (PM, BNB) and ten interviewers, who were generally medical residents (at least in their 6th year), students in biological curriculum or nurses, from Bangui and Brazzaville Universities. All the interviewers were trained during three weeks in the main assessment and the physical measures included in the protocol. Interviewers were selected according to their performances during the training.

Groups of three interviewers were supervised on the field by the project coordinator or an epidemiologist during the whole duration of the study. Field interviews were randomly supervised to control the good execution of the different tasks, and solve any problem when occurring.

### Data management

The interviewers collected data directly onto laptop computers during the study. Questionnaires were computerized using EpiData (version 3.1) software, including conditional skips and interactive checking for data consistency. Identical Epidata files were used in each setting, ensuring facilities to data processing and merging. The 10/66 algorithm was processed using SPSS (version 17.0, IBM SPSS Statistics). Data cleaning, and processing of variables was done using Stata (version 10.1, StataCorp, College Station, Texas, United States) and SAS (version 9.2, SAS Institute, Cary, NC) softwares.

Datasets are stored on secured servers at Limoges University, and declared to the ‘Commission Nationale de l’Informatique et des Libertés’ (CNIL) according to our national regulations on personal data.

### Ethical issues

Each participant and family members were informed about the study and consent was obtained from subject and/or family when the former was unable to express his/her consent. Written consent was obtained every time when feasible. For illiterate people, the purpose of the study was verbally explained and the consent was obtained by thumbprint marks.

In both countries, ethical committees, supervised by Ministry of Public Health in CAR and the CERSSA (Comité d’Ethique de la Recherche en Sciences de Santé) in ROC, approved the study protocol, as well as the CPP-SOOM-IV (Comité de la Protection des Personnes Sud-Ouest Outre-Mer) in France.

### Statistical analyses

For every site, participants’ characteristics will be described: age, sex, marital status, education level, and health status. Continuous variables will be reported using means and standard deviations (SD) for the normally distributed variables otherwise medians and inter-quartile ranges (IQR) will be reported. Categorical variables will be reported using numbers and percentages. Normality of the variables will be examined using standard test with skewness and appropriate transformations applied if required. Chi-square, Fisher exact or Student’s t-tests will be used to determine if statistically significant differences exist between groups, according to the nature of the variable and numbers.

The prevalence of DSM-IV and 10/66 dementias, AD and MCI, by gender and age in 5-years bands will be estimated for each site, with their 95% confidence intervals. We will standardize for age and sex the prevalence rates on the Sub-Saharan population (United Nations Department of Economic and Social Affairs Population Division) to make comparisons of dementia according to the 10/66 and the DSM-IV criteria between our study sites. Clinical severity for dementia cases will be described. The independent effects of age, sex, education and ApoE genotype on dementia prevalence will be provided from a Poisson regression model, separately for each site and then combined.

Multivariate regressions analyses will be used to estimate risk associations between the different exposures and cognitive status (dementia, MCI, cognitively normal), adjusted on the relevant socio-demographic, vascular, psychosocial factors, or other exposure measured during the assessment.

## Discussion

### Methodological issues

One-phase studies may be an ideal for dementia prevalence studies (Prince 2000) but two-phase studies, often preferred in low and middle-income countries, are a more pragmatic approach to research when carrying out a full clinical assessment of large populations is not possible due to practical and financial constraints.

Two-phase surveys are known to increase the risk of attrition between the first and the second phase (Prince 2000), consequently increasing the risk of moving, death or refusal from people with dementia before to establish the diagnosis. However, previous 2-phase studies in Benin, Central African Republic, Congo and Tanzania (Guerchet et al. 2009; Paraïso et al. 2011; Guerchet et al. 2010; Paddick et al. 2013) showed lower attrition rates than described by the 10/66 Dementia Research Group (Prince 2000), reassuring us about the feasibility and the robustness of two-phase epidemiological surveys in SSA. The threshold used to select participants for the second phase and clinical assessment was furthermore defined according to sensitivity and specificity found in previous studies carried out in the same elderly populations. Another advantage of a two-stage design is to exclude from the diagnosis of Dementia, transient delirium.

As our programme also aimed at determining the neuropsychological profiles of people with dementia within this context, a more comprehensive interview with a clinician/specialist and the inclusion of additional neuropsychometrical tests (Free and Cued Selective Reminding Test, Isaac’s Set Test of verbal fluency and Zazzo’s cancellation task) was indicated for the second phase.

While dementia defined by the 10/66 criteria has shown higher prevalences of dementia across the 10/66 studies, authors suggested that the DSM-IV may consistently underestimate the prevalence of dementia by including only the more severe cases, especially in regions with low awareness and less developed (Llibre Rodriguez et al. 2008). Recently, the age-standardized dementia prevalence estimates according to those two different criteria, DSM-IV and 10/66, were reported and compared in a rural area of Tanzania (Paddick et al. 2013). Prevalence of dementia was again higher when using the 10/66 criteria than the DSM-IV. Of note education was a significant predictor of dementia when defined by the 10/66 criteria, but not of DSM-IV criteria. However, the authors did not conclude whether the association between education and diagnosis was authentic or the result of an educational bias within the diagnostic instrument. As we included the GMS-AGECAT and the modified-10 word list in addition to the CSI-D in our programme, the 10/66 dementia prevalence will be estimated, allowing further comparisons between both DSM-IV and 10/66 dementia criteria, and the potential effects of education.

During this programme, both urban and rural areas were investigated enabling to have a larger picture of the burden of dementia in those countries. Sampling methods were yet different. A sampling proportional to the size of the main subdivision of each capital was applied in both urban areas. Indeed in both capitals distribution of ethnic groups differs from one subdivision to another. The definition of a catchment area within the city would not have permitted to recruit a sample of participants fully representative of the Bangui and Brazzaville’s inhabitants. Furthermore, door-to-door studies in defined catchment areas of both cities have been carried out previously (Guerchet et al. 2010). An exhaustive sampling, by a door-to-door knocking, was preferred in rural areas. This procedure was more suitable in areas where addresses are completely inexistent, and will allow us to tag the participants for further studies more easily. Data collection in a geographically defined area was also financially and logistically (i.e. blood sample procedure) feasible. However, as distribution of ethnic groups differs from one region to another in both countries, we are aware that the results reported for rural areas would not necessarily be generalizable to any other rural area within the country.

### Feasibility

The feasibility of this programme is an essential issue, and mainly raises the two following points: security in the countries/areas where the research is carried out and reliability of our network/collaboration.

The ROC and the CAR have known political disturbances and civil wars in the 90s and early 2000s. Since, the situation is stable and secure in the ROC. Some areas were still hold by rebels in eastern and northern CAR, but Nola and Bangui were completely secured during the time we were planning and carrying out the surveys there. Nevertheless, the coordinators and the teams respected all the usual safety rules and procedures (Governments of CAR and ROC as well as French Embassies/Diplomacies).

As already mentioned in the preparation section, the local collaborators and the French research units have been collaborating on previous projects on dementia before to take on this new programme, which ensured us the quality and strengths of this multicentre collaboration. Furthermore, the two local collaborators (BNB and PM) are associated members of the Inserm UMR1094 (Limoges) and have carried out several other research projects on the epidemiology of epilepsy or other neurological disorders.

### Dissemination

Results from this programme will be disseminated at both international and national levels. They will be communicated to international agencies of concern, in particular to the World Health Organization (African office, Brazzaville) and Alzheimer Disease International (ADI, London). These findings will be used in each country to raise public awareness, stimulate clinical training and practice, and influence social and health care policies.

A strategy for dissemination was established between all the investigators during a meeting held in Limoges in May 2013. Dissemination through peer-reviewed articles will be prioritized. All the investigators have access to the full anonym database. The final dataset will be approved by the coordinator of the programme and stored securely on Limoges University network. All proposals for publications should be addressed and approved by the coordinator and all the investigators. Public access to the database by external investigators will be opened one year after the release of the final version (i.e. 01/07/2015). Applications for use of the data should be sent to the responsible of this programme (preux@unilim.fr or ient@unilim.fr).

### Role of the funding source

This study was funded by a grant from the French National Research Agency (ANR-09-MNPS-009-01).

The sponsors of the study had no role in study design, data collection, data interpretation, or writing of the report. All authors had full access to all the data in the study, and the corresponding author had final responsibility for the decision to submit for publication.

## Abbreviations

10/66 DRG: 10/66 Dementia Research Group
ABI: Ankle-Brachial Index
AD: Alzheimer disease
ADI: Alzheimer’s Disease International
AGECAT: The automated geriatric examination for computer assisted taxonomy
AMC: Arm muscle circumference
APOE: Apolipoprotein E
APP: Amyloid beta precursor protein
BMI: Body mass index
BPSD: Behavorial and psychological symptoms of dementia
CAR: Central African Republic
CERAD: Consortium to establish a registry for Alzheimer’s disease
CERSSA: Comité d’Ethique de la recherche en sciences de santé
CNIL: Commission nationale de l’Informatique et des Libertés
CPPSOOM: Comité de protection des personnes sud-ouest outre-mer
CRP: C-Reactive protein
CSI-D: Community screening interview for dementia
DBP: Diastolic blood pressure
DNA: Deoxyribonucleic acid
DPD: Dependent personality disorders
DSM: Diagnostic and statistical manual of mental disorders
EDTA: ethylenediaminetetraacetic acid
EDAC: Epidemiologie des Démences en Afrique Centrale
EPIDEMCA: Epidemiology if dementia in Central Africa
GMS: Geriatric mental state
GWAS: Genome Wide Association Studies
HIC: High income countries
ICD: International classification of diseases
IQR: Interquartile range
LMIC: Low and middle income countries
MCI: Mild cognitive impairment
MUAC: Mid-upper arm circumference
NINCDS-ADRDA: National Institute of Neurological and Communicative Disorders and Stroke and the Alzheimer’s Disease and Related Disorders Association
NPI: NeuroPsychiatric inventory
PCR: Polymerase chain reaction
PS1: Presenilin-1
RGPH: Recensement de la Population et de l’Habitat
ROC: Republic of Congo
SBP: Systolic blood pressure
SD: Standard deviation
SOF: Study of osteoporotic fracture
SSA: Sub-Saharan Africa
WHO: World Health Organization
WHR: Waist hip ratio.
