# Erratum to: Epidemiology of dementia in Central Africa (EPIDEMCA): protocol for a multicentre population-based study in rural and urban areas of the Central African Republic and the Republic of Congo

**DOI:** 10.1186/s40064-016-2094-8

**Published:** 2016-05-05

**Authors:** Maëlenn Guerchet, Pascal Mbelesso, Bébène Ndamba-Bandzouzi, Sophie Pilleron, Ileana Desormais, Philippe Lacroix, Victor Aboyans, Pierre Jésus, Jean-Claude Desport, Achille E. Tchalla, Benoît Marin, Jean-Charles Lambert, Jean-Pierre Clément, Jean-François Dartigues, Pierre-Marie Preux, for the EPIDEMCA Group

**Affiliations:** INSERM UMR 1094, Tropical Neuroepidemiology, Faculty of Medicine, 2 rue du Docteur Marcland, 87025 Limoges, France; University Limoges, School of Medicine, Institute of Neuroepidemiology and Tropical Neurology, CNRS FR 3503 GEIST, Limoges, France; King’s College London, Centre for Global Mental Health, Institute of Psychiatry, Health Service and Population Research Department, London, UK; Department of Neurology, Brazzaville University Hospital, Brazzaville, Republic of Congo; Department of Neurology, Amitié Hospital, Bangui, Central African Republic; CHU, Department of CardioVascular Surgery, Limoges, France; Department of Cardiology, Dupuytren University Hospital, Limoges, France; CHU, Department of Nutrition, Limoges, France; EA 6310, Disability, Activity, Aging, Autonomy and the Environment (HAVAE), Limoges, France; CHU, Department of Medical Information and Evaluation, Clinical Research and Biostatistic Unit, Limoges, France; INSERM U744, Institut Pasteur de Lille, Lille, France; Hospital and University Federation of Adult and Geriatric Psychiatry, Limoges, France; INSERM U897, Victor Segalen Bordeaux II University, Bordeaux, France

## Erratum to: SpringerPlus (2014) 3:338 DOI 10.1186/2193-1801-3-338

There is a typo in Table 1 the original version of this article (Guerchet et al. 2014). The number of blood samples for Rep. of Congo/Gamboma (rural) was ‘836’, while it should be ‘436’. This has been corrected in Table 1.

**Table 1:** EPIDEMCA population-based survey, Central Africa, 2011–2012

Country	Study area	Sample	Interviews completed	Response rate %	Blood sample	Lost-of follow-up	Start/end
Central African Rep.	Nola (rural)	501	473	94.41	393	66	Nov. 2011/Feb. 2012
	Bangui (urban)	514	500	97.28	441	48	Jan. 2012/March 2012
Rep. of Congo	Gamboma (rural)	561	529	94.30	436	93	Aug. 2012/Dec. 2012
	Brazzaville (urban)	537	500	93.11	409	17	Sept. 2012/Nov. 2012
